# Supplementary material for: Porcine Circovirus Type 2 Induces Single Immunoglobulin Interleukin-1 Related Receptor (SIGIRR) Downregulation to Promote Interleukin-1β Upregulation in Porcine Alveolar Macrophage
Source: Viruses. 2019 Nov 3;11(11):1021. doi: 10.3390/v11111021 (PMC6893714; doi:10.3390/v11111021)
Supplement: Supplementary file 1 [file viruses-11-01021-s001.pdf]

Table s1. Differentially expressed (absolute fold change ≥2and *P < 0.05*) inflammatory cytokines and receptors in PCV2-infected PAMs.

Gene Symbol	Description	1 HPI (Fold Change)	24 HPI (Fold Change)	48 HPI (Fold Change)	Refseq
BMP2	Aminoacyl tRNA synthetase complex-interacting multifunctional protein 1	2.15	-2.27	-1.45	NM_001195399
C5	Complement component 5	-1.18	2.08	3.09	NM_001001646
CCL1	Chemokine (C-C motif) ligand 1	15.81	-4.32	-2.22	NM_001166491
CCL17	Chemokine ligand 17-like protein	1.29	1.25	-5.63	NM_001256147
CCL2	Chemokine (C-C motif) ligand 2	9.55	2.05	-3.65	NM_214214
CCL20	Chemokine (C-C motif) ligand 20	20.81	10.12	12.27	NM_001024589
CCL21	Chemokine (C-C motif) ligand 21	3.05	-4.79	-3.33	NM_001005151
CCL22	C-C motif chemokine 22-like	-1.73	-1.22	-5.34	NM_001256776
CCL3L1	Chemokine (C-C motif) ligand 3-like 1	51.60	-1.48	-6.82	NM_001009579
CCL4	Chemokine (C-C motif) ligand 4	109.09	-1.06	-3.46	NM_213779
CCL5	Chemokine (C-C motif) ligand 5	3.63	-1.26	-1.19	NM_001129946
CCL8	Chemokine (C-C motif) ligand 8	24.18	3.82	-2.85	NM_001164515
CCR2	Chemokine (C-C motif) receptor 2	-4.27	-1.75	-1.42	NM_001001619
CCR7	Chemokine (C-C motif) receptor 7	3.00	-1.94	-2.32	NM_001001532
CD70	CD70 molecule	9.84	-1.94	1.08	NM_001044531
CSF1	Colony stimulating factor 1 (macrophage)	2.19	-4.91	-19.38	NM_001244523
CSF2	Colony stimulating factor 2 (granulocyte-macrophage)	203.09	5.29	4.99	NM_214118
CSF3	Colony stimulating factor 3 (granulocyte)	13.89	5.28	9.22	NM_213842
CXCL10	Chemokine (C-X-C motif) ligand 10	1.36	-1.49	-28.25	NM_001008691
CXCL2	Chemokine (C-X-C motif) ligand 2	53.05	13.85	10.37	XM_005652553
CXCR2	Chemokine (C-X-C motif) receptor 2	-1.26	3.26	4.83	XM_005672195
CXCR4	Chemokine (C-X-C motif) receptor 4	2.68	3.70	4.64	NM_213773
IL10	Interleukin 10	6.82	5.93	5.98	NM_214041
IL10RA	Interleukin 10 receptor, alpha	1.95	1.54	3.74	XM_003129890
IL12B	Interleukin 12B (natural killer cell stimulatory factor 2, cytotoxic	1.29	2.15	3.07	NM_214013

	lymphocyte maturation factor 2, p40)				
IL13	Interleukin 13	3.58	1.19	1.98	NM_213803
IL15	Interleukin 15	-4.41	-1.33	-1.29	NM_214390
IL16	Interleukin 16	-2.09	1.16	1.19	NM_213751
IL18	Interleukin 18 (interferon-gamma-inducing factor)	2.55	3.15	5.69	NM_213997
IL1A	Interleukin 1, alpha	197.54	10.07	6.99	NM_214029
IL1B	Interleukin 1, beta	367.77	10.19	7.81	NM_214055
IL1RN	Interleukin 1 receptor antagonist	-1.28	-1.85	-3.62	NM_214262
IL23A	Interleukin 23, alpha subunit p19	52.69	6.52	4.38	NM_001130236
IL27	Interleukin 27	11.36	1.22	1.14	NM_001007520
IL-5	Interleukin 5	2.36	1.17	1.06	NM_214205
IL6	Interleukin 6 (interferon, beta 2)	3.27	10.00	2.27	NM_214399
IL7	Interleukin 7	1.28	2.08	1.63	NM_214135
IL7R	Interleukin 7 receptor	10.12	7.58	7.23	NM_001146128
IL8	Interleukin 8	27.59	5.39	2.42	NM_213867
LIF	Leukemia inhibitory factor (cholinergic differentiation factor)	24.81	-1.78	1.91	NM_214402
IL33	Interleukin-33-like	1.69	3.18	5.11	XM_003121912
LOC100519468 (TNFSF14)	Tumor necrosis factor ligand superfamily member 14-like	1.36	-2.10	-3.57	NM_001260482
LTB	Lymphotoxin beta (TNF superfamily, member 3)	2.12	-2.52	1.44	NM_001185138
NAMPT	Nicotinamide phosphoribosyltransferase	2.03	1.13	-1.08	NM_001031793
SPP1	Secreted phosphoprotein 1	-1.10	-2.16	-8.29	NM_214023
TNF	Tumor necrosis factor	146.29	1.94	1.77	NM_214022
TNFRSF11B	Tumor necrosis factor receptor superfamily, member 11b	-1.92	-5.28	-1.98	XM_003481346
TNFSF10	Tumor necrosis factor (ligand) superfamily, member 10	-2.82	3.97	13.31	NM_001024696
TNFSF13B	Tumor necrosis factor (ligand) superfamily, member 13b	1.31	2.79	3.23	NM_001097498

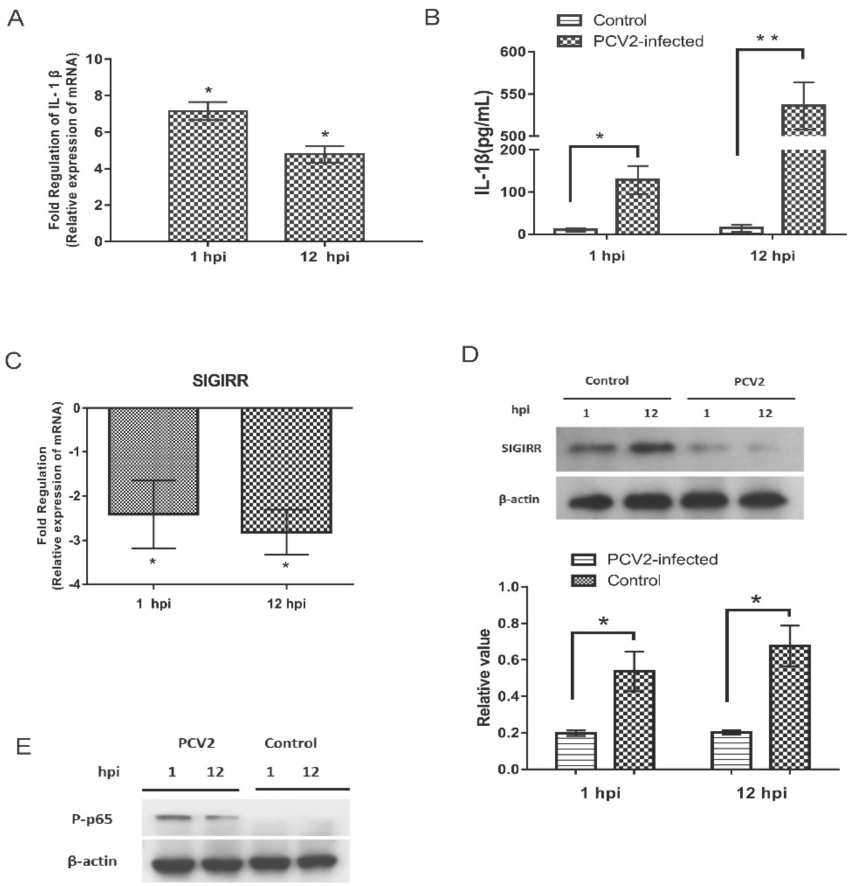

Figure s1. The expression of IL-1β, SIGIRR, and the activation of NF-κB in 3D4/2 post PCV2 infection. 3D4/2 cells were infected with PCV2b with MOI of 1 and 12h. (**A**) The IL-1β mRNA levels were detected by qPCR in 3D4/2 at 1 and 12 hpi. (**B**) The IL-1β secretion was measured in 3D4/2 at 1 and 12 hpi using a commercial porcine IL-1β ELISA kit. (**C**) The expression of SIGIRR in 3D4/2 at 1 and 12 hpi were examined by qPCR. The expression of SIGIRR mRNA was normalized to actin-β and presented as fold change relative to the control. (**D**) The expression of SIGIRR in protein level was detected by Western blot. Densitometric analysis was performed to quantify the ratio of SIGGIR to actin-β. Results are mean ± SE of three independent experiments. Statistical differences in data were evaluated by Student's t-test. * represent $p < 0.05$, ** represents $p < 0.01$. (**E**) The p-p65 was detected by Western blot in PCV2-infected 3D4/2 at 1 and 12 hpi.
